# Solid–Water Interface Interaction of Selenium with Fe(II)-Bearing Minerals and Aqueous Fe(II) and S(-II) Ions in the Near-Field of the Radioactive Waste Disposal System

**DOI:** 10.3390/ijms24010315

**Published:** 2022-12-24

**Authors:** Michaela Matulová, Eva Duborská, Peter Matúš, Martin Urík

**Affiliations:** 1 Radioactive Waste Repository Authority (SÚRAO), Dlážděná 6, 11000 Prague 1, Czech Republic; 2 Institute of Laboratory Research on Geomaterials, Faculty of Natural Sciences, Comenius University in Bratislava, Mlynská Dolina, Ilkovičova 6, 84215 Bratislava, Slovakia

**Keywords:** selenium, ferrous minerals, nuclear waste, sorption, reduction processes

## Abstract

Selenium can be highly toxic in excess for both animals and humans. However, since its mobile forms can be easily adsorbed with ferric minerals, its mobility in the natural oxic environment is generally not an issue. Still, the removal and immobilization of the long-lived radioactive isotope ^79^Se from the contaminated anoxic waters is currently a significant concern. ^79^Se can be accessible in the case of radionuclidesˈ leaching from radioactive waste disposals, where anoxic conditions prevail and where ferrous ions and Fe(II)-bearing minerals predominate after corrosion processes (e.g., magnetite). Therefore, reductive and adsorptive immobilizations by Fe(II)-bearing minerals are the primary mechanisms for removing redox-sensitive selenium. Even though the information on the sorptive interactions of selenium and Fe(II)-bearing minerals seems to be well documented, this review focuses specifically on the state of the available information on the effects of the redox properties of Fe(II)-bearing solid phases (e.g., ferrous oxides, hydroxides, sulfides, and carbonates) on selenium speciation via redox transformation and co-occurring coprecipitation.

## 1. Introduction

Selenium is essential for organisms, yet it is highly toxic in excess. Therefore, the evaluation of factors affecting its environmental mobility and bioavailability represents an urgent issue for environmental toxicologists [1,2,3,4].

The redox chemistry of selenium is complex since it naturally appears in the oxidation states of −II, 0, IV, and VI. Selenides and elemental selenium usually have low solubility and insignificant mobility, while selenites and selenates, which are abundant in oxic environments, are considered highly soluble and reasonably migrant [5,6,7,8]. Therefore, selenium mobility largely depends on the oxidation state, thus, it is important to consider the redox sensitivity of its species.

Besides the six stable natural isotopes, there is also a long-lived radioisotope ^79^Se, which occurs as a fission product; and its only probable long-term source to the environment is the leakage from radioactive waste repositories. The primary natural geogenic sources of stable selenium isotopes include organic-rich sedimentary rocks, phosphatic rocks [9,10], carbonate rocks, and soluble salts in marine sediments [11,12]. Thus, its sources in the environment are mainly excavated rocks from coal mining, tunnel construction, and underground space development; however, it is also released by coal combustion, nonferrous metal smelting, and agricultural runoffs [13,14,15,16].

High-level radioactive wastes (HLW) and spent nuclear fuel (SNF) are disposed of in a multi-barrier system with a series of engineered and natural barriers that work together to protect people and the environment. Engineered barrier systems are technical/artificial components consisting of waste forms, waste canisters, buffer, backfill material, and seals. Natural barriers represent host rock and overlying formations. In this review, we focused on the scenario that water would enter a container and radionuclides would be leached. We used this scenario with the aim to discuss the effectiveness of iron minerals in immobilizing selenium oxyanions in wastewater systems, using information from research focused on radioactive waste disposal.

Regarding the issue of radionuclide mobilization from nuclear waste (e.g., ^79^Se), the interaction of aqueous selenium with Fe(II)-bearing minerals has received extensive attention in recent years. This is because it is expected that the steel or copper containers used in the nuclear waste or spent nuclear fuel containment (exemplified in Table 1) will corrode in time, which can result in the intrusion of groundwater into the nuclear waste package, thus, increasing the probability of selenium radioisotope transportation into the environment and biosphere. In the case of steel containers, the corrosion process eventually leads to the formation of various Fe(II)-bearing minerals depending on the container’s material, type of buffer, and host clay composition [17,18,19,20,21]. Notably, magnetite, green rust, mackinawite, pyrite, and siderite are considered abundant and typical Fe(II)-bearing corrosion products appearing in natural aquifers as well as on engineered barriers [19,21,22,23,24,25,26,27,28,29].

Geological disposal of SNF and HLW in deep clay formations will ultimately lead to the dissolution and slow leaching of soluble redox-sensitive radionuclides (such as ^79^Se), which is expected to leach at least partially as selenium oxyanions. Although part of the leached selenium could sorb on corrosion products of the steel canisters, the remainder will reach the surrounding clay host rock [30]. The reducing environment in this clay host rock and possible microbial presence might change its speciation and transport behavior. A good understanding of the retention and reactivity of selenium in the presence of microorganisms is therefore required, although it was not the focus of this study it is briefly discussed.

Sulfides exist in a fracture and a surrounding host rock. It is possible that along a flow path from a fractured rock, sulfide dissolved in groundwater starts to migrate into a bentonite layer. If sulfides are present in the repository, microbiologically influenced corrosion can contribute to spent fuel container corrosion [31]. For corrosion of copper via sulfide, corrosion is highly dependent on the presence of sulfide species. The only meaningful concentrations that may occur will do so because of the remote microbial activity of sulfate-reducing bacteria (SRB). Sulfide is a potential corroding agent to copper. In the case of steel canisters, SRB influence iron corrosion by releasing free Fe(II) ions into the solution surroundings. The SRB produce H_2_ in the process of formation of iron sulfide, FeS [32,33].

Under revealing reducing conditions in a near-field area of a deep repository for spent fuel, the oxidation states of selenium −II and 0 predominate. However, with oxidation agents, which might be carried with groundwater, selenium can be oxidized. It was also noted that selenium occurred in the oxidation state of –II in an investigated spent fuel sample, replacing oxygen atoms in a disordered UO_2_ lattice [34]. Still, in highly radioactive waste concentrates, selenium was also discovered in its mobile oxidation state of IV [35]. The neutral to alkaline conditions typical for the deep geological disposal sites for SNF and HLW reduce the efficiency of surface adsorption of negatively charged radionuclides, such as ^79^Se, onto the host rocks and containing materials. Therefore, under these conditions, reductive immobilization is considered the most effective immobilization mechanism for selenium oxyanions in comparison to surface adsorption. It enables the precipitation of new, relatively insoluble, solid phases [36]. To accurately study these processes in detail, various standard and innovative instrumental analysis and techniques have been employed, including X-ray diffraction (XRD), X-ray photoelectron spectroscopy (XPS), X-ray absorption spectroscopy (XAS), vibrating sample magnetometry (VSM), Raman and IR spectroscopy, as well as Mössbauer spectrometry.

The geochemical behavior of selenium radioisotopes regarding their redox transformations constitutes a priority research area for the agencies in charge of safety assessment, especially, since there is a possibility that a significant part of the ^79^Se inventory in spent nuclear fuel is readily accessible to aqueous leaching in the repository environment of radioactive waste [37]. Therefore, this review summarizes the results of investigations regarding the redox and sorptive interactions between selenium and Fe(II)-bearing minerals, which have been reported up to the summer of 2022. The utilization of findings and outcomes for selenium removal from wastewater is also discussed.

## 2. Interaction of Selenium with Fe(II)-Containing Oxides and Hydroxides

Magnetite and green rust are structurally mixed-valent iron oxides and hydroxides, respectively, containing both Fe(II) and Fe(III) in the brucite-like layers along with intercalated anions. Magnetite is a black ferromagnetic mineral. It is a member of the spinel group of minerals, while green rust is a layered double hydroxide. The latter can be alternated with interlayer anions (e.g., SO_4_^2−^, CO_3_^2−^, and Cl^−^), forming various types of green rust, such as hydroxysulfate, hydroxycarbonate, and hydroxychloride [38]. The reactivity of both mixed-valent iron minerals in aqueous solutions strongly depends upon their ability to undergo redox transformations [39]. Redox-sensitivity of magnetite also manifests in the formation of magnetite–maghemite solid solution, commonly referred to as nonstoichiometric or partially oxidized magnetite. Its stoichiometry, with respect to the Fe(II)/Fe(III) ratio, strongly influences several chemical and physical properties, including coercivity, sorption capacity, and reduction potential. Since the increase in activity of H^+^ only slightly promotes the dissolution of magnetite and while the co-occurring decrease in Fe(II)/Fe(III) ratio is largely consistent with the reduced sorption capacity, Cheng et al. [40] concluded that the magnetite stoichiometry seems to be the major factor affecting its binding capabilities. Gorski and Scherer [41] reported that the doping of magnetite with Fe(II) increased its stoichiometry due to reduction of the octahedral Fe(III) atoms in magnetite. This phenomenon alters the bulk redox properties of the particle to make the reduction of the contaminants more favorable.

The self-induced redox potential reported for magnetite reduction to aqueous Fe(II) ions varies between +0.38 V at pH 3 and +0.27 V at pH 7 [42]. However, the determination of magnetite’s redox potential is not a trivial issue, especially since the low concentrations of dissolved Fe(II) ions can alter the reductive potential of magnetite significantly. This is also complicated by the fact that the redox potentials of solid oxides also vary with the crystal phase and grain size [43]. Furthermore, magnetite comprises two redox-active sites, which both contribute to the net measured potential simultaneously [44]. Thus, due to variations in the Fe(II)/Fe(III) ratio, alterations in the apparent redox potential of magnetite can occur [45]. Therefore, conflicting magnetite redox potentials at neutral pH have been reported, including values of +0.27 V [42], –0.31 V [43], and –0.38 V [46]. There is a relation between the redox potential and magnetite stoichiometry (Fe(II)/Fe(III) ratio) which should be considered for magnetite’s successful application as a redox-active remediation agent for environmental pollutants [47,48], including selenium.

As noted previously, dissolved Fe(II) is often reported as a catalyst enabling selenite reduction by other more potent electron donors [49]. In the radioactive waste repository, Fe(II) is formed after the elemental iron of the steel container reacts with the hydrogen proton and water. Then, under acidic conditions, FeSe_2_ precipitates can be formed in the presence of selenide [50], or via the redox transformation of selenium oxyanions or elemental selenium in the presence of redox-active Fe(II)-bearing minerals. However, it was reported that at dissolved selenium activities over the 10^−6^ mol⋅L^−1^ and reducing conditions, the elemental selenium stability covers a wide range of pH. Below this activity the conversion to selenide or selenite is favorable only in highly alkaline solutions [51]. Since selenides are thermodynamically stable only under strongly reducing conditions, the elemental selenium reduction should be governed by strong reducing agents and, thus, involvement of Fe(II) is unlikely. However, there is a unique biological mechanism adapted by Se-respiring bacteria (e.g., *Bacillus selenitireducens*), which enables dissimilatory reduction of elemental selenium to selenide [52], but also can reduce both selenate and selenite to elemental selenium that is accumulated extracellularly [53]. This indicates that the common occurrence of metal selenides in sedimentary rocks can be the outcome of microbiological activity rather than purely from chemical processes [54].

The near-neutral pH and anaerobic conditions, which are usually characteristic for radioactive waste repositories, enable the formation of Fe(II)-hydroxide, a metastable phase, that easily transforms into the (redox-active) magnetite through the Schikorr reaction [55,56]. Under strongly alkaline conditions, Fe(OH)_2_ or Fe(OH)_3_ can be formed [50].

The pH and resolving corrosion product indeed affect the selenium redox transformation/immobilization, especially since its redox sensitivity seems to be influenced by the degree of protonation under the prevailing acid–base balance in the solution. Since the standard reduction potential of Fe(III)/Fe(II), as reported by Shi et al. [57], is slightly higher (+0.771 V) than the one of the H_2_SeO_3_/Se(0) couple (+0.740 V), the reduction of selenite by Fe(II) ions in aqueous solutions should not be thermodynamically favorable [58]. However, the reported formal potentials of HSeO_3_^−^/Se(0) and SeO_3_^2−^/Se(0) reach the values of 0.903 V and 0.875 V, respectively [59,60], which makes the coupled redox transformation of selenite and Fe(II) ions in aqueous solutions feasible. It suggests that the selenite reduction is governed by its protonation in aqueous solutions and, thus, by the solution’s pH. Therefore, the ability of Fe(II) ions to reduce selenite has not been demonstrated in some instances. Such is in the case of Shi et al. [57], who did not detect selenite reduction when the pH of the solution was below 5.6, even with significant excess of Fe(II) ions. On the contrary, Finck and Dardenne [58] at an Fe(II):Se(IV) molar ratio of 4:1 using XAS noted the formation of elemental selenium under anoxic conditions with the final pH and E_h_ of 2.84 and +0.165 V, respectively (the presence of mineral phases was not confirmed).

Missana et al. [61] suggested that the reason for not always successfully observing selenium reduction, depends on the different reaction conditions. The difference in results was probably caused by the lower solid-to-liquid ratio, a smaller mean particle diameter with a higher surface area, and a smaller initial selenium concentration. Since electron transfer from Fe(II) to selenite is strongly favored by heterogeneous surface reaction, while reduction is restricted in solution [62,63], the lack of reduction in the system is most likely due to the inhibited surface reaction because of the much smaller available surface area per anion. The smaller surface area of the magnetite used might be one of the reasons why reduction has not been observed in the investigated systems. Furthermore, when small initial concentrations are used, it is also necessary to pay attention to the resolution ability of the device.

In the case where the iron oxides and hydroxides contain Fe(II) ions (e.g., magnetite, green rust, or Fe(II)-hydroxide), they are capable of catalyzing the reduction of selenium oxyanions to produce non-soluble elemental selenium and/or iron selenides [30,64,65,66,67]; several are listed in Table 2 with their respective thermodynamic parameters. In the case of Fe(II)-bearing minerals, it was reported that such minerals (e.g., green rust, magnetite, and mackinawite) reduce at least 89% of selenite within a day [68]. For instance, the reduction of SeO_4_^2–^ by Fe(II)-hydroxide can be expressed as follows (1, 2) [69]:

Fe(OH)_2_ + H_2_O ⇔ Fe(OH)_3_ + H^+^ + e^−^ (1)

SeO_4_^2−^ + 2H^+^ + 2e^−^ ⇔ SeO_3_^2−^ + H_2_O (2)

Furthermore, iron oxides can have an important role in abiotic reduction of selenium in sediments as a field study by Chen et al. [70] suggested. Selenite can be abiotically reduced to elemental selenium. However, this reduction process appears to be mediated by active surfaces.

It has been suggested that green rust exchanges its interlayer anions for selenate and slowly reduces the selenate to elemental selenium via electron transfer from structural Fe(II) ions, but so far, only Cl-green rust [72] and SO_4_-green rust [73] reactions with selenate have been reported with vastly different selenium uptake kinetics. However, green rust has rarely been observed in natural environments due to its meta-stability concerning magnetite and siderite. Thus, the interaction between green rust and selenate is very unlikely in such an environment. On the other hand, magnetite is a relatively stable iron oxide, and thus, as highlighted in Table 3, it is expected to be formed as a steel canister corrosion product under anoxic and highly saline conditions [18,19,26]. Still, a SeO_4_-green rust compound that is isomorphous to SO_4_-green rust can be formed in the presence of Fe(OH)_2_ as reported by Refait et al. [74], confirmed by XPS. There, the iron hydroxide precipitation, the simultaneous accumulation of SeO_4_^2−^ anions, and the redox transformations of both iron and selenate inside the solid phase allow the formation of this unique green rust. It is hypothesized that no oxidizing agent other than selenate itself is needed in this process. Furthermore, these selenium species were found to be less mobile, when partially bound to iron compounds, and/or forming iron salts.

Skovbjerg et al. [75] suggested that selenite is reduced to elemental selenium, mainly at the edges of green rust. Although, simultaneously, green rust is oxidized to goethite, and there was no evidence of selenite entry into the green rust interlayers. Atomic force microscopy images showed tiny particles at the edges of the green rust crystals and the growth layers. Grey precipitates remained at the water/air interface in the samples, while it suggested a particle size in the nanometer range. These visible by-eye, extremely fine precipitates were only present at the beginning of the reactions and had disappeared by the time elemental selenium could be detected with XRD. Therefore, it could indicate that these particles were amorphous selenium phases that became crystalline as they grew. The tiny particles that initially precipitated on the edges of green rust particles could be this amorphous selenium. Still, it could also be ferrihydrite, formed from fast reprecipitation of the iron from dissolved, oxidized green rust. This is also probable because goethite was observed with transmission electron microscopy and XRD.

**Table 3:** Corrosion experiments under oxic and anoxic conditions on various steels with identified corrosion products using spectroscopic methods.

Oxygen Conditions	Material	Experimental Conditions	Methods	Corrosion Products	Literature
Anoxic conditions	Copper-coated steel	Anoxic and aerated-to-anoxic conditions in 3 mol⋅L^−1^ NaCl	SEM, SEM-EDS, and Raman spectroscopy	Maghemite, akaganeite, lepidocrocite and goethite	Standish et al. [76]
	Carbon steel	Na_2_CO_3_ ∕ NaHCO_3_, Na_2_SO_4_, and NaCl solutions simulating concentrated groundwaters at pH 8.9 on the composition of the corrosion products formed on carbon steel at room temperature	in-situ Raman spectroscopy	Iron carbide, carbonate-containing, and to a lesser degree, sulfate-containing green rusts, and magnetite	Lee et al. [19]
	Carbon steel	Artificial Swedish granitic groundwater (diluted sodium/calcium chloride solutions) at pH 8, temperatures in the range of 30–85 °C and pressures at 1, 10, and 100 atm	XRD	Iron oxides (magnetite)	Smart et al. [26]
Oxic conditions	Low-carbon steel	Chloride, and sulfate ions in bicarbonate and phosphate aqueous solutions	Micro-Raman spectroscopy	Green rust generated in bicarbonate or phosphate solution containing chloride and/or sulfate ions	Simard et al. [77] and [78]
Various atmospheres	Carbon steel and weathering steel e	A tmospheric exposure	XRD and Raman spectroscopy	Lepidocrocite, goethite, and magnetite	Antunes et al. [79]
Oxic conditions	Carbon steel	Exposed to weathering	SEM, SEM-EDS, FTIR	Goethite	Costa et al. [80]

Hayashi et al. [81] investigated removal of selenate by hydroxysulfate green rust using XAS analysis. Selenate ion was reduced to elemental selenium at pH 9.0, whereas the existence of a small amount of intermediate selenite was detected at pH 7.5. Comparing the mass balance of the amount of consumed ferrous iron in SO_4_-green rust, and the amount of selenate removed from the liquid phase, ferrous ions were consumed approximately six times more than selenate ions in mol units. This is also indirect but convincing evidence that the oxidation of Fe(II) in SO_4_-green rust leads to the simultaneous reduction of selenate to elemental selenium. XRD analysis showed that the final product of SO_4_-green rust depends on the pH: magnetite for pH > 9.0, goethite for pH < 8.0, and their mixture at pH 8.5. These results indicate that the solution pH has a significant effect on the reaction path of selenate removal by SO_4_-green rust.

Börsig et al. [82] investigated the selenium speciation during the magnetite formation and performed detailed analyses of the solid phases using K-edge X-ray absorption near-edge structure (XANES) and extended X-ray absorption fine-structure (EXAFS). Their results showed that the coprecipitation process via interaction with Fe(II)-hydroxide and green rust caused the reduction of selenium under anoxic conditions during the early phase. The early selenium interaction led to the formation of a nanoparticulate FeSe. However, with the progression of the aquatic system oxidation, it was oxidized and transformed into gray trigonal elemental selenium. The results suggested that the selenium is retained regardless of whether the oxidation of the unstable iron oxides forms pure magnetite or other iron mineral phases (e.g., goethite).

Onoguchi et al. [67] recently suggested that the reduction of selenate into selenite and elemental selenium was catalyzed by green rust-sulphate through two different mechanisms. First, after the adsorption on a solid phase (homogeneous redox reaction) and second, without adsorption at a solid–liquid interface (heterogeneous redox reaction), where the dissolved selenate could be reduced upon contact with the green rust-sulphate. Furthermore, the removal of the selenate involved a redox reaction with green rust-sulphate at both pH 8 and 9, although the redox reaction is more pronounced at pH 9. The results of Onoguchi et al. [67] indicated that the selenite is immobilized by simple adsorption at pH 8. However, the adsorption process is followed by the reduction step at pH 9, and elemental selenium is formed. During this process, green rust is oxidized to goethite or magnetite. The rate parameters also suggested that after the green rust is oxidized, the immobilization of selenium becomes slower. Furthermore, the analysis of Onoguchi et al. [67] using X-ray adsorption fine structure (XAFS) also suggested that the adsorbed selenate was reduced to selenite and/or elemental selenium through a parallel reduction pathway. In other words, the adsorbed selenite cannot be reduced further to elemental selenium, probably due to the depletion of Fe(II) in the proximity of the Fe(III) adsorption sites.

Kim et al. [83] observed using XANES and EXAFS that selenite is transformed to elemental selenium or selenide on the magnetite surface as it encounters reducing conditions at E_h_ of –0.27 V. Their results indicate that elemental selenium is directly accumulated in the mineral phase. In contrast, adsorbed selenide can undergo a subsequent comproportionating reaction with selenite in solution to form elemental selenium. If reduced species are exposed to oxidizing conditions (+0.44 V), remobilization via oxidation to selenite is possible. Even though the formation of selenate from oxidation of the lower valence forms can be mediated by magnetite, there was no available spectroscopic evidence for further oxidation in solution.

The ferrous oxides seem to have efficient retardation properties for the higher oxidation states of selenium. However, for the most reduced selenium species, selenide, the K_d_ values in the range of 5.4 × 10^−4^ to 1.1 m^3^ kg^−1^ for ferrous oxide and 6.8 × 10^−4^ to 5.6 × 10^−2^ m^3^ kg^−1^ for magnetite at pH 7–13 were obtained. The sorption data and the triple layer surface complexation model (TLM) calculations suggest that the dominant sorption mechanisms of selenide were inner-sphere complexation (≡FeSe−) for ferrous oxide but outer-sphere complexation (≡FeOH_2_+−HSe−) for magnetite [84]. 

## 3. Interaction of Selenium with Ferrous Sulfides

In terms of selenium interaction with dissolved sulfide in water systems, Jung et al. [85] hypothesized that H_2_S was the main sulfide species that reacted and precipitated with the selenite. Since the reported average pKa values of hydrogen sulfide are 7.01 and 13.78 [86], and no precipitates were formed in the sulfide solution at pH 11 (investigated by XPS), Jung et al. [85] concluded that neither HS^−^ nor S^2−^ were the active species that reacted with the selenite to form selenium-containing precipitates.

However, Kharma et al. [87] noted that during the selenite reduction, besides the dissolved H_2_S, the partially (de)protonated species could also initiate a complex interchalcogen redox cascade that involves the synthesis of various transient and reactive sulfur and selenium species. Their speculative model introduced a series of nucleophilic substitution and reductive elimination reactions resulting in the formation of reactive species such as HSSe(O)SH, HSSeSH, Se_n_S_8−n_, HSSH, H_2_S_x_, and H_2_Se. This process is compelling for both biological and environmental studies on selenite. It suggests that the abiotic transformation of selenite results in the synthesis of reactive species that affects the (intra)cellular metabolism and enables selenite removal from water [88].

Geoffroy and Demopoulos [89] studied the interaction of selenite with sodium sulfide from weakly acidic solutions. Below a pH of 7.0, the precipitation reaction was complete; however, when the pH increased to values between 7.0 and 9.5, the precipitation of selenium was incomplete. Furthermore, above pH 9.5, the solution turned dark red, but no precipitation appeared. At these pH values, the reaction between selenium and sodium sulfide was complete in less than 10 min. The orange “selenium sulfide” precipitates, characterized using XRD, scanning electron microscopy (SEM), and chemical analysis, were crystalline in the form of aggregated dense particles with their sulfur/selenium molar ratio varying from 1.7 to 2.3. The precipitate was Se–S solid solution consisting of ring molecules of the following Se_n_S_8−n_ formula, where n = 2.5–3.

Pettine et al. [90] noted that the reduction of selenite was indeed controlled by the speciation of dissolved hydrogen sulfide in natural waters under hypoxic and anoxic conditions. However, their investigation of selenite (using hydride generation atomic absorption spectrometry) reduction kinetics in a (dissolved) sulfide solution also highlighted varying degrees of selenite species involvement in the process. Regarding the recommended pKa values of 2.70 and 8.54 for selenous acid [51], the major contributors in the acidic solutions seemed to be H_2_SeO_3_ which reacted with H_2_S neutral species. On the other hand, the effect of HSeO_3_^−^ on the reduction rate, which reacted with HS^−^, was pronounced under neutral and alkaline conditions.

There is huge role of indirect selenium immobilization in nature (e.g., in lake sediments) by sulfides produced by microbiological activity. Previous research [89,91,92,93] maintained the role of the abiotic reduction of selenite to elemental selenium by sulfides at environmental concentration levels. Zehr and Oremland [91] and Oremland et al. [92] suggested that the relative abundance of elemental selenium occurring in the surface sediments of the Kesterson National Refuge (USA) could involve an indirect reduction of selenite by hydrogen sulfide produced by SRB rather than a direct biological reduction of selenite and selenate by SRB.

The reduction of selenite and selenate occurs by Desulfovibrio desulfuricans, which is a sulfate-reducing bacterium that produces spherical (selenium, sulfur) sub-micro particles outside the cell. Depending on the medium composition the formed particles can be crystalline or amorphous. Furthermore, amorphous-like Se-rich spherical particles may also occur inside the bacterial cells. The bacteria are more active in the reduction of selenite than selenate. The Desulfovibrio desulfuricans bacterium can extract sulfur in the solid solution particles and transform S-rich particles into Se-rich and selenium crystals. Photoautotrophs, such as Chromatium spp., can oxidize sulfide (S^2−^). When the bacteria grow in sulfide- and selenide-bearing environments, they produce amorphous-like globules inside the cells [94].

The selenite transformation was also allowed in the presence of ferrous sulfidic minerals, e.g., mackinawite (FeS_1−x_, 0 < x < 0.07, tetragonal), pyrite (FeS_2_, cubic) and pyrrhotite (Fe_1−x_S, 0 < x < 0.125, monoclinic and hexagonal) under conditions characteristic for anoxic subsurface environments and aquatic sediments. There, the non-oxidative proton-promoted dissolution of some ferrous sulfidic minerals can promote the generation of H_2_S [95], which can directly reduce the aqueous selenite in the acidic solution [96] and form discrete surface precipitates. Alternatively, the ferric sulfide minerals have been identified as having active surface sulfhydryl functional groups that can transform redox-sensitive elements. There is evidence that mackinawite at pH 4.0–6.0 produces elemental selenium, Fe_7_Se_8,_ and FeSe after its interaction with the selenite [63]. Han et al. [97] also noted using XPS for investigation that during the oxidation of mackinawite, the surface sulfide was involved in the selenium reduction resulting in the formation of polysulfides or elemental selenium.

Naveau et al. [98] demonstrated using XPS and XANES the presence of reduced selenium species on the pyrite (and chalcopyrite) surfaces, which suggests a redox process coupled with the sorption. Furthermore, the selenium reduction occurred concomitantly with the oxidation of pyritic sulfur. It was noted that iron (and copper) were not involved in the redox process. However, the formation of FeSe_2_ was more probable in the synthetic solid phases, while the surface complexation or ionic exchange processes were more likely prevalent on the natural mineral surfaces. Wang et al. [99] also investigated the immobilization of selenium by pyrite and its release from the mineral’s surfaces using in situ scanning electrochemical microscopy. They observed that immobilized selenium was in the form of elemental selenium and FeSe_2_. While the elemental selenium was the primary form adsorbed onto pyrite surfaces, the abundance of selenium associated inhomogeneously with sulfur sites of pyrite was scarce. Similarly, Charlet et al. [100] proposed using XANES-EXAFS spectroscopic techniques that the selenium interaction with pyrite resulted in the precipitation of elemental selenium and FeSe_2_. While at pH ~5.9 and ~6.2, the ratio of elemental selenium and FeSe_2_ was approximately 3:7, it became approximately 1:1 at pH over 8.0.

While performing the experimental observations under oxygen-free (<1 ppm) conditions, Kang et al. [101] noticed the release of Fe(II), which was partitioned between the bulk solution and pyrite surface at pH ≈ 4.5 and 4.8, with the Fe(II) ion density at the pyrite-solution interface about four orders of magnitude higher than that in the bulk solution. The precipitation of iron oxyhydroxide at pH ≈ 6.6 was noted, resulting in the decrease of free dissolved iron which prevented the formation of FeSe_2_. This was revealed with XANES spectroscopic measurements performed at the Se K-edge (12.658 keV). It was concluded that the Fe(II) ion mediated electron transfer enables the selenite reduction by pyrite, thus, the solution pH and concentration of iron have a significant effect on the selenite reaction rate and the reaction product. The involvement of iron in the selenite reduction contradicted the previously mentioned study of Naveau et al. [98].

Finck et al. [102] investigated selenide retention by adsorption and coprecipitation with mackinawite performed by multi-edge XAS investigation. Unlike selenium in higher oxidation states, selenide had no significant influence on the morphology and structure of the mackinawite precipitates. Furthermore, selenium was situated in a mackinawite-like sulfide environment, which suggested a highly reactive mackinawite dynamical dissolution/recrystallization mechanism. Xiong et al. [50] reported that under reducing conditions, elemental selenium is either incorporated into pyrite within the mineral phase or forms the mineral ferroselite (FeSe_2_). Liu et al. [103] studied the capacity of pyrite to immobilize selenide using in situ XANES technique. After only several minutes of reaction, they observed that at least 97% of the selenide initially present in the solution disappeared and was immobilized as elemental selenium at the pyrite surfaces. However, no elemental selenium was noted in the study of Yang et al. [104], who investigated the surface properties of pyrrhotite (Fe_1-x_S) exposed to an alkalic solution (pH 8) of selenide. Since no changes were found in the XRD patterns after modification, it was concluded that the mineral core remained intact, and impregnation allowed the formation of a 20–30 nm thick amorphous Se-S-Fe shell.

Diener and Neumann [105], investigated the uptake of selenide by iron sulfides synthesized in batch experiments under anoxic conditions. Results from XRD and SEM analyses revealed the formation of pyrite and mackinawite. They discovered an inhomogeneous selenium distribution with a higher accumulation in the center of the pyrite grains measured with focused ion beam analysis. This was probably due to the progressive depletion of selenium from solution regarding sulfur.

The selenide did not show a significant impact on the structural changes after its interaction with ferrous sulfides. However, mackinawite and pyrite are subjected to transformations when interacting with selenium oxyanions. For example, the more thermodynamically stable pyrite was formed because of partial mackinawite oxidation at the surface of the sulfur sites [58]. However, the role of the surface-active sulfhydryl group is more complex. In the case of the metastable sulfur-deficient mackinawite mineral, its surface contains strongly acidic mono-coordinated and weakly acidic tri-coordinated sulfurs, which determine the acid–base and surface charge, respectively [106]. Furthermore, at acidic conditions (pH ≤ 3), mackinawite is dissolved after its reaction with selenite, and H_2_S is generated. However, at pH above 3, S^2-^ is produced [107,108]. The results suggest that pyrite and its most important precursor phase, mackinawite, are efficient in removing selenium from solution, which may contribute to reducing the mobility of ^79^Se released from radioactive waste.

## 4. Interaction of Selenium with Ferrous Carbonates

Calcite is relatively abundant in the earth’s crust, and it is one of the most common minerals on the earth’s surface [109,110]. Furthermore, calcite plays an essential role in nuclear waste storage environmental systems and surroundings in several countries, e.g., the Czech Republic and Finland. In the geochemical systems, due to their buffering effects, carbonates influence the properties of soils and sediments as well as the chemistry of the waters [110,111]. Pure calcite usually does not contain Fe(II) ions but it is possible under certain conditions. Calcite and its modifications are not considered important scavengers for radionuclide ^79^Se in underground systems where the ^79^Se can be potentially leached from the nuclear waste [112,113]. However, calcite dissolution (3) is an important process that can change groundwater parameters and, thus, affects the sorption capacity of iron-bearing minerals as well as their stability and crystallization. Wijnja and Schulthess [114] noted that the concentration of CO_3_^2−^ up to 0.2 mmol⋅L^−1^ promotes selenate adsorption onto iron oxides in the pH range of 6 to 8, most likely via alteration of the surface protonation and charge. However, above this concentration, the sorptive interactions become competitive.

CaCO_3_ ⇔ Ca^2+^ + CO_3_^2−^ (3)

Calcite’s dissolution rate in aqueous solutions is highly dependent on pH. While at low pH (<4), the dissolution rate is almost directly proportional to the H^+^ concentration, the dissolution rate is independent of the H^+^ content at pH above 5.5 [115]. According to Appelo and Postma [116], the (unstable) molecule of H_2_CO_3_ forms in solution at pH < 6.3, and at a pH above 10.3 the carbonate (CO_3_^2−^) dominates, with bicarbonate (HCO_3_^−^) being prevalent in between.

Calcite can be formed by processes of microbial-induced precipitation via naturally occurring bacteria binding soil particles together through calcite precipitation. The main groups of microorganisms that can induce carbonate precipitation are photosynthetic microorganisms such as cyanobacteria and microalgae; sulfate-reducing bacteria; and some species of microorganisms involved in the nitrogen cycle [117].

Calcite shows a high ability to incorporate magnesium to form magnesium calcite (MgCO_3_) and dolomite (CaMgCO_3_) [118]. However, in the case of corrosion, Fe(II) accumulation, and reactivity, the siderite (FeCO_3_) precipitation is relatively slow, even when the solution is supersaturated. Furthermore, the precipitation rate decreases as the pH decreases. It was observed that equilibrium was achieved after several months at around neutral pH, while it was reached even more slowly in acidic solutions. The dissolution rate can be even further reduced in environments containing sulfates or orthophosphates [119]. Moreover, equilibration with amorphous ferric oxyhydroxides in oxidizing waters, with amorphous ferric oxyhydroxides and siderite in slightly reducing waters, and with siderite and amorphous ferrous sulfide in strongly reducing waters, controls the dissolved iron concentrations [120].

Furthermore, carbon species, the product of calcite dissolution, can occur in groundwater systems in gaseous form such as CO_2_, or dissolved in water as CO_2_, H_2_CO_3_, HCO_3_^−^, and CO^2−^. They have effect on many geochemical processes such as acid–base reactions (pH-regulating), dissolution and precipitation of minerals, metal-complexation reactions, and adsorption processes [121,122]. Carbonates have a marginal effect on selenium interaction with iron. It was discovered that selenite sorption onto goethite was reduced only in the case that carbonate concentration was more than 1000 times that of selenite [123].

Natural siderite is considered an effective reactive barrier in selenite-polluted environments due to its high sorption capacity for selenite. Furthermore, it comprises structural Fe(II) whose presence can immobilize selenium via reduction to elemental selenium. Thus, siderite can contribute to a safer environment by immobilizing radioactive elements leached from nuclear waste, such as isotope ^79^Se, and delay their appearance in the biosphere. It was suggested that during the storage of HLW, the corrosion of iron envelope most likely results in the formation of a siderite or chukanovite (Fe_2_(CO_3_)(OH)_2_) [22,23,24]. The spontaneous direct precipitation of siderite requires anaerobic conditions and substantial supersaturation. It most likely involves the highly pH dependent homogenous nucleation of amorphous Fe(II)-carbonate, which than recrystallizes to either metastable chukanovite or siderite [124]. The siderite stability area is small (although it can be underestimated) since it dissolves below a pH value of 6.2, while the increase in solution pH (over the value of 9) and E_h_ transforms siderite into an Fe(II)-hydroxide and different iron oxides (e.g., hematite), respectively [125].

Pavón et al. [28] emphasized the importance of siderite occurring in the geological barrier regarding selenium immobilization. Expectedly, the sorption capacity of siderite for selenite ions was higher than that of other iron minerals, including magnetite, goethite, and hematite. Chakraborty et al. [126] suggested that the increasing amount of Fe(II) ions, which coprecipitated with the calcite, increased the sorption of selenite compared to that of Fe-free calcite at pH 7 under anoxic conditions. Nearly half of the total sorbed selenite was reduced to elemental selenium by Fe(II) ions within a day in the form of needle-shaped red monoclinic precipitates located at the calcite’s surfaces, as was suggested from XANES results. Scheinost and Charlet [63] also confirmed with XAS analyses the efficient reduction of selenium to its elemental state on freshly synthesized siderite surfaces within a day. Badaut et al. [127] studied controlled electrochemical redox reactions between aqueous selenite and siderite by in situ XANES monitoring. Similar to previous studies, their results confirmed that more than 60% of initial selenite was reduced on the siderite surfaces after 20 h. They also proposed that the reaction included probably both selenite coprecipitation with Fe(II) and sorption onto generated Fe(III) colloids.

It was hypothesized that the immobilization process of selenite by siderite includes at least three different mechanisms, all appearing simultaneously. The first involves selenite coprecipitation with dissolved Fe(II) ions, while minerals such as FeSeO_3_ are formed [62,128]. The second mechanism describes selenite adsorption onto the surfaces of Fe(III)-hydroxide colloids, which precipitate after Fe(II) oxidation [129,130]. The last mechanism involves direct adsorption of selenite onto the siderite surfaces with or without reduction. Moreover, selenite sorption via carbonate substitution may occur, which is similar to reported selenite sorption onto calcite surfaces [127]. This mechanism was also confirmed by Yokoyama et al. [131] who showed that selenite can be incorporated into calcite by substituting the carbonate and that a slight expansion of crystal volume could accommodate the geometric incompatibility of selenite. This conclusion was confirmed using both, EXAFS spectroscopy and quantum chemical calculations.

Unfortunately, we found only a few published works on the interaction between mobile radionuclides and chukanovite (Fe_2_(CO_3_)(OH)) because this mineral was identified only recently [132]. Thus, the mobility of electrons in their structure or reactivity towards other contaminants is not currently well known. Kirsch et al. [133] studied the reaction of plutonium with chukanovite under reducing conditions using Pu L_3_-edge XANES and EXAFS spectra. They speculated that the partial reduction of Pu(V) to Pu(III) by chukanovite occurred, but this was not confirmed by using supportive thermodynamic calculations with spectroscopic data. However, no investigations on its interaction with mobile selenium radionuclides, such as ^79^Se, are currently available.

## 5. Practical Implications of Ferrous Minerals for Selenium Removal

Based on the previous reports, selenium immobilization from water resources using Fe(II)-bearing minerals seems sensible to mitigate human and ecosystem exposure to selenium at potentially toxic concentration levels in contaminated areas. Compared to other techniques, the immobilization of pollutants onto the surfaces of Fe(II)-based adsorbents has the advantage of high efficiency, low cost, and easy operation [134,135,136]. Furthermore, the inclusion of Fe(II)-bearing minerals in the composite sorbents (e.g., biosorbents) is viable since it can enhance the affinity of composites towards the selenium, especially in the case of selenium removal from radioactive wastes or waters primarily contaminated with other inorganic pollutants. For instance, organic coatings may enhance the performance of Fe(II)-bearing minerals since they combine the assemblages of nanometer-sized minerals and organic components that further increase the range of practical applications of composites; e.g., Zhang et al. [137] utilized granular activated carbons for iron oxide coating achieving the promising 97.3% selenite removal from mine waters.

Magnetite is relatively efficient in selenium immobilization, and its sorptive performance can also be further enhanced with coatings applied to its surfaces. Goberna-Ferrón et al. [30] studied the effects of silica surface coatings on magnetite redox-catalytic ability toward selenium oxyanions. Electron transfer still occurred even after partial oxidation of iron oxide nanoparticles and even under different silica surface coverages. Their results revealed the presence of selenite and elemental selenium in the solid residues after the sorption. However, the abundance of elemental selenium was lower in the case of the reaction with selenate compared to selenite, most likely due to the reduction of selenate to selenite being the rate-limiting step. Still, the iron oxides were shown to be influential collectors of selenite and selenate via adsorption processes and reductive precipitation even when modified with coatings.

A mixture of various non-ferrous oxides with ferrous salts also showed promising results in the removal of selenium. Suzuki et al. [138] studied the immobilization of selenate using agents prepared by mixing ferrous salts with MgO. It was observed that the selenate was not effectively immobilized until the ferrous salt was present. Thus, it is very likely that selenate was reduced to elemental selenium or selenide by Fe(II) ions. The magnetite was found to be the final product of the reaction. Similarly, Tian et al. [139] studied the immobilization of selenate using CaO and MgO mixed with ferrous salt. Almost all of the selenate was reduced to selenite in the CaO-based reaction within 7 days. Then, the generated selenite was mainly sorbed onto the precipitated iron-based minerals, e.g., Fe_2_O_3_ and FeOOH. However, reducing the selenate to selenite with a MgO-based reaction was less efficient. Furthermore, as for the associations of selenium in the solid residue, the majority of selenium species were preferentially distributed onto the Mg(OH)_2_ through outer-sphere adsorption.

The other promising method for wastewater treatment is the use of synthesized layered double hydroxide (LDH) nanoparticles [140,141]. For instance, Fe-Ti layered double hydroxide was successfully used for selenite removal from marine sediments and soils [142]. Recently, three types of Mg(II)-Fe(III) pyroaurites and Fe(II)-Fe(III) green rust layered double hydroxides were studied for selenite and selenate removal. The anion exchange was confirmed as an important uptake mechanism by chloride pyroaurites and sulfate pyroaurites. Sulfate pyroaurites appeared to have a greater uptake capacity at high selenium concentrations, while chloride pyroaurite was more efficient at lower selenium concentrations. Because the latter outcome was more relevant to common environmental and wastewater conditions, chloride pyroaurite is also preferable for water treatment applications. On the other hand, the carbonate form of green rust showed only minimal selenate removal efficiency.

## 6. Conclusions

This review summarizes the abiotic and briefly biotic processes of aqueous selenium transformation and immobilization in conjunction with Fe(II)-bearing minerals to provide fundamental information regarding the prospects of ferrous ion and mineral utilization in selenium removal from contaminated waters. Even though it may seem that the current state of this issue is well-researched, there is still a lack of reliable and comprehensive data analysis that would provide sensible outcomes on the interaction of low-valanced selenium species (e.g., selenide) with Fe(II)-bearing minerals under anoxic conditions. Moreover, the surface interactions of selenium with some Fe(II)-bearing minerals (e.g., chukanovite) have not been studied even today. Nevertheless, some general conclusions could be drawn, e.g., selenite and selenate can be effectively reduced and immobilized onto mineral surfaces, either forming FeSe or FeSe_2_ depending on the stability and redox properties of Fe(II)-bearing minerals and the conditions under which the removal is examined. The processes of reductive immobilization of selenium by Fe(II)-bearing minerals can slow the extension of selenium contamination into the environment. This is an urgent issue, especially in the case of intrusion of radionuclides from radioactive waste disposal, including selenium. Furthermore, the implication of ferrous ions appears to be seen as having a resurgence in environmental applications. Nevertheless, several research gaps and challenges are needed to be addressed to further advance the knowledge on the removal of selenium species by ferrous minerals.

## Figures and Tables

**Table 1:** Comparison of individual deep storage concepts for SNF and HLW and discussed engineering barriers in selected countries.

Country	Examples of Canister Material for Spent Nuclear Fuel	Host Rock
Finland	Copper	Crystalline rock
Sweden	Copper	Crystalline rock
France	Steel	Clay
Switzerland	Steel	Clay
Canada	Copper clad steel	Crystalline rock, sediments
Germany	Pollux	Not decided yet
Belgium	Steel	Clay
South Korea	Copper	Crystalline rock, sediments
Czech Republic	Steel	Crystalline rock

**Table 2:** The thermodynamic data for several iron selenides (T =298.15 K, p = 0.1 MPa) [71].

Compound	$\Delta_{f}H_{m}^{0}$ (kJ·mol^–1^)	$S_{m}^{0}$ (J·K^–1^·mol^–1^)	$C_{p,m}^{0}$ (J·K^–1^·mol^–1^)
δ-Fe_0.875_Se			
FeSe_2_ (cr)	− 108.7 ± 15.0	86.8 ± 1.0	72.9 ± 1.0 (c)
β-Fe_1.04_Se	− 69.6 ± 4.0	72.1 ± 0.8	57.1 ± 0.7 (c)
γ-Fe_3_Se_4_	− 235.0 ± 30.0	279.8 ± 3.0	220.1 ± 2.0
α-Fe_7_Se_8_	− 463.5 ± 20.0	613.8 ± 5.0	442.1 ± 4.0

$S_{m}$ —(molar) entropy, $C_{p.m}$—(molar) heat capacity at constant pressure, $H_{m}$—(molar) enthalpy, ^0^—superscript for the standard state. Abbreviations are used as subscripts ∆ to denote the type of chemical process.

## Data Availability

Not applicable.
